# The influence of local officials’ promotion incentives on Public-Private Partnership project investment from a political-performance-driven perspective: Empirical evidence from China 

**DOI:** 10.1371/journal.pone.0337404

**Published:** 2026-01-02

**Authors:** Lisha Zhang, Mingyang Yue, Shasha Du, Rong Zheng, Tao Zhu, Boyuan Tang

**Affiliations:** 1 School of Marxism, Hubei Communications Technical College, Wuhan, Hubei, People’s Republic of China; 2 Department of Economics, Jiangsu Administration Institute, Nanjing, Jiangsu, People’s Republic of China; 3 School of Social and Public Administration, Lingnan Normal University, Zhanjiang, People’s Republic of China; Bangladesh Sericulture Research & Training Institute, BANGLADESH

## Abstract

The investment in PPP projects in China is large but quite uneven. Based on the political economy theory, using data from the public‒private partnership (PPP) industry issued by local municipal authorities from 2014 to 2018 in China, this paper explains the phenomenon of uneven PPP investment from a politically performance-driven perspective. The study shows that local officials have a strong incentive to increase investment in PPP projects that have positive visual effects on performance appraisal. Driven by promotion incentive, PPP investment demonstrates a phenomenon of “emphasizing performance over livelihood” and “emphasizing aboveground over underground”. The correlation between promotion incentives for municipal party secretaries and the annual incremental investment amount in growth-based PPP categories has an inverted U-shaped curve. Furthermore, the age of mayors has a heterogeneous influence on PPP investment, with those under 52 years of age being more willing to increase investment in growth-based PPP categories, as well as growth-based-visible PPP categories. Our study explores the political reasons for project investment mismatch and reveals the negative effect of local officials’ promotion incentives, which provides empirical experience in China for other countries to explore the relationship between officials’ promotion incentives and project investment.

## 1. Introduction

To guide the healthy development of the PPP model throughout recent decades, China’s State Council, Development and Reform Commission, and Ministry of Finance have issued a series of guidance documents in an effort to regulate the public–private partnership (PPP) industry, prevent and control invisible risks, and rationalize the use of financial funds. However, when we check the investment status of PPP projects in China, we find an interesting phenomenon: the investment in PPP projects is quite uneven.

According to the PPP Information Platform in the China Public Private Partnerships Center (which is abbreviated as CPPPC), by the end of 2019, 6383 PPP projects had entered the implementation phase. Among these projects, the total number of PPP categories in municipal engineering, transportation, and comprehensive urban development was 3965, which exceeded 60% of the total PPP number. The total investment amount of these three PPP categories accounted for 74.73% of the total PPP investment amount. In contrast, the total number of PPP categories in education, health care, tourism, culture, sports, pensions, and social security was only 927, accounting for 14.52% of the total PPP number, and the total investment amount of these seven PPP categories accounted for 6.83% of the total PPP investment amount. Although uneven investment in PPP projects is a frequent phenomenon in all countries, the high polarization of PPP projects in China makes us wonder if there is a specific reason for this phenomenon [1].

The PPP failure rate, support from government policies and local economic and social development are important factors that affect PPP investment [2]. Compared with local economic and social development, the PPP failure rate and support from government policies are more strongly connected with government political behavior [1,3]. On the basis of the data statistics of China’s full-calibrated PPP exit rate in the CPPPC, from 2014 to 2018, the exit rates of PPP categories in municipal engineering, transportation and comprehensive urban development are all low to below 40%; the exit rates of PPP categories in pension, social security, tourism and health care are all as high as 50%. The PPP exit rate is inversely related to the PPP investment share, which suggests that government political behaviors play an important role in the PPP selection process.

An analysis of PPP data from the CPPPC revealed that there is an uneven investment in PPP projects in China. On the basis of political economy theory, we explain the phenomenon of uneven PPP investment from a politically performance-driven perspective. Encouraged by promotion incentives, local officials prioritize industries that contribute to the local economy to enhance their performance [4]. Hence, local officials make considerable efforts to increase PPP investment, especially PPP investment, which could benefit their political performance [3]. However, the PPP model, which aims to improve the efficiency of public service supply and alleviate the pressure of local government debt, should not be abandoned as a tool for local officials to seek political performance and gain political promotion [5]. If this occurred, it would be necessary to identify the underlying reason and determine the solutions.

PPP projects that entered the implementation phase from 2014 to 2018 were used as research objects for this paper to investigate the impact of key local officials’ promotion incentives (mayors and municipal party secretaries) on PPP investment. This paper divides PPP categories into growth-based categories and livelihood-based categories and divides growth-based categories into growth-based-visible categories and growth-based-invisible categories. It uses tenure and age as promotion incentive indicators, predicts local officials at different tenures and ages facing different promotion incentives, and examines the effect of promotion incentives on uneven PPP investment. This paper aims to explore the political reasons for project investment mismatch, identify the negative effect of local officials’ promotion incentives, and provide Chinese solutions for this mismatch phenomenon.

The marginal contribution of the paper lies in two points. First, it combines the theoretical foundations of political economy with the external factors – performance drive and official promotion- that affect PPP project investment, analyzes the reasons for uneven investment in PPP projects from a performance-driven perspective. Second, considering the promotion system of local officials in China, it provides empirical experience for uneven investment in PPP projects, and make policy recommendations for the standardized appraisal mechanism of officials from the aspect of correcting distortion of public resources allocation.

## 2. Literature review and hypothesis proposal

Political environmental elements such as the institutional environment, government intervention, and governance ability are key factors that influence the success of PPP [6–8]. Main and Mukand [9] suggest that politicians provide public goods or services to citizens, whereas citizens vote in elections on the basis of the performance of politicians. It is difficult for citizens to see or judge the performance of politicians on the basis of certain public goods or services that are difficult to observe. To gain more support from voters and maximize political interests, politicians tend to choose to provide those public goods or services that are easy to observe. Zhou [10] provides a Chinese interpretation of the political performance drive: the promotion of local officials in China comes from the appraisal by superiors, and a promotion tournament system based on GDP growth makes the promotion of local officials depend on a number of measurable economic indicators. Stimulated by the promotion incentive, local officials give more weight to industries that contribute to the local economy to enhance their performance. Moreover, because the central government assigns higher-level officials to inspect local areas, higher-level officials may judge local officials’ political performance on the basis of what they see. To obtain a better inspection appraisal, local officials are naturally inclined to support industries that have a strong visual impact. This Chinese interpretation provides a theory for research on the influence of local officials’ promotion incentives on technological innovation, bond issuance and environmental protection [11–14].

On the discussion of the bias to PPP investment, the scale of project, the structure of risk and the cost of project are the reasons for the uneven PPP investment [1]. The investors are cline to invest to the PPP project with small scale and short period [15,16]. The PPP project with a smaller predictable risk gets more chance to receive investment [17,18]. Hoppe & Schmitz, Iossa & Martimort, and Martimort & Menezes [19–21] discussed the selection of PPP projects from the comparative perspective of cost and risk. As local governments’ decision is the key factor to the implementation of PPP project, PPP investment implies the local officials’ behavior motivation [22]. Debt risk has received more attention in the study of the connection between official promotion and PPP projects. Local governments have an incentive to use the PPP model to hide government debt [23–25]. Considering the stimulating effect of local officials’ performance pressure on the PPP investment [26], obtaining promotions is one of the major reasons for the alienation of PPP projects into the tool for the government to issue local debt illegally [27]. As PPP projects are beneficial to the acceleration of economic growth, local officials are willing to take excessive risks to push the implementation of PPP projects when the promotion incentive is high enough [3].

Accordingly, we propose Hypothesis 1:

H1. Driven by political promotion, local officials have a strong incentive to increase investment in PPP projects.

From an objective perspective, as economic performance is a key indicator for local officials’ promotion, the accomplishment of economic target has direct connection with the effect of promotion incentive [28]. Closely related to the literature on the negative effects of GDP-oriented promotion [10], those studies show that in pursuit of regional economic performance, local officials neglect public services such as education, health care, and environmental protection [29,30]. Based on the different contribution of PPP projects to local economic growth, the investment on PPP projects are classified to into economic preferences and social preferences [31]. Local officials have strong incentives to increase the input of “productive” and “economic” public goods while ignoring the input of “nonproductive” and “social” public goods [32,33]. Therefore, the promotion incentive characterized by political performance drive has encouraged local officials to commit more resources to PPP projects that have a close connection to the local economy while neglecting livelihood PPP projects (e.g., education, culture, and elderly care).

Accordingly, we propose the first sub-sections of Hypothesis 1:

H1a. Local officials prioritize growth-based PPP investment over livelihood-based PPP investment.

From the subjective perspective, in order to gain good impression, politicians tend to choose to provide those public goods or services that are easy to observe [9]. Although there are strict disciplines to constraint the tours of inspection in China [34], the local officials are still with some hopes to make good impression to the superiors through grand constructions [4]. Thus, the political inspection system exacerbates PPP polarization, with PPP projects providing aboveground facility construction (e.g., rail transportation, town development, etc.) receiving more resources, while PPP projects providing underground facility construction (e.g., sponge cities, water supply and heating, etc.) receiving fewer resources.

Accordingly, we propose the second sub-sections of Hypothesis 1:

H1b. Local officials prioritize aboveground PPP projects over underground PPP projects.

Previous studies have also discussed the relationship between promotion incentives and economic performance. The performance improvement increases the probability of promotion, but it shows a nonlinear relationship [35]. To increase economic performance and increase the probability of promotion, local officials have a strong incentive to expand their investment in urban infrastructure [36] while neglecting to invest in livelihood public goods such as education, health care and environmental protection [37,38]. Tenure and age are the main indices used to measure officials’ promotion incentives [39,40]. When officials’ tenure and age approach the promotion thresholds, they may feel greater promotion pressure and are more eager to push local economic growth through credit and investment [41,42]. Wu and Zhou [4] verified the impact of promotion incentives on urban maintenance and construction from a public goods visibility perspective and noted that this impact shows an inverted U-shaped trend with variations in tenure and age.

Accordingly, we propose Hypothesis 2:

H2. There is a threshold in promotion incentives. Because of this promotion threshold, there is an inverted U-shaped curve between local officials’ promotion incentives and PPP investment.

**Fig 1:**
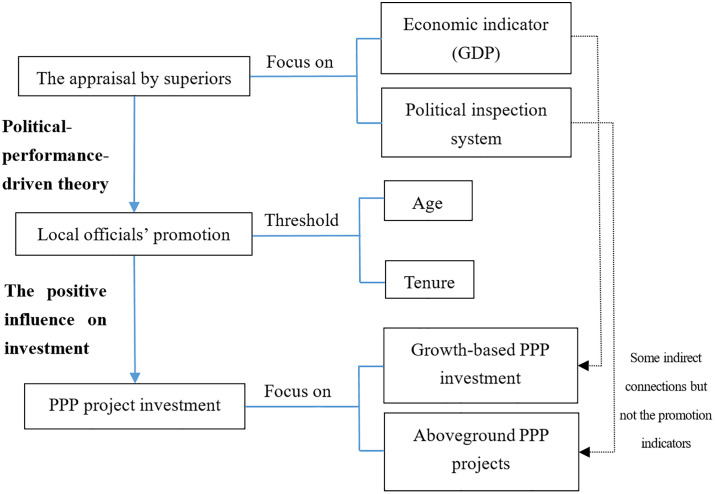
The flowchart of mechanism analysis.

Fig 1 is the flowchart of mechanism analysis.

## 3. Materials and methods

### 3.1 Sample selection and statistical description

The data of the sample consists of three sections: PPP projects, local official characteristics and city characteristics. From 2014 to 2018 in China, PPP projects experienced explosive growth and received less regulation. It is a typical period in which local officials increase PPP investment driven by promotion incentives. To exclude the external influence of strict regulation and obtain robust and significant results, we set the sample period as 2014–2018. We clarify that the data of annual incremental PPP projects in 2014 correspond to the data of the characteristics of officials in 2014, and the corresponding relationships remain the same every year.

#### 3.1.1 Classification of PPP categories.

Data on PPP categories is collected on the PPP Information Platform in the CPPPC. The PPP samples include all annual incremental PPP projects from 2014 to 2018. Excluding the projects that directly belong to the provincial level, autonomous prefectures and autonomous regions, as well as the projects that have been removed from the PPP Information Platform, 6003 sample projects match the information of local officials.

All projects are divided into growth-based categories and livelihood-based categories. Growth-based categories tend to contribute to local economic construction, whereas livelihood-based categories tend to provide social public goods or services for daily life [30–33]. The growth-based categories include both growth-based-visible categories and growth-based-invisible categories. Growth-based-visible projects are closely linked to urban construction aboveground which are easily to observe, whereas growth-based-invisible projects are mostly built underground and difficult to observe [4].

Transportation, government-subsidized housing projects, comprehensive urban development, municipal engineering (public transport, squares, rail transit, landscaping, municipal roads, car parks) and government infrastructure are growth-based-visible categories. Municipal engineering (power supply, gas supply, heat supply, water supply, pipe networks, sponge city construction, refuse disposal, drainage, sewage treatment and others) and water conservancy are growth-based-invisible categories. Education, tourism, social security, sports, culture, elderly care, science and technology, energy, ecological construction and environmental protection, health care, forestry and agriculture belong to livelihood-based categories. The number of PPP samples and the investment amount for each category by year are shown in Table 1.

**Table 1:** Statistical description of the PPP samples’ number and investment amount by year.

	Number (pcs)						Investment amount (billion yuan)					
	2014	2015	2016	2017	2018	Total	2014	2015	2016	2017	2018	Total
Growth-based categories	135	1007	1316	1483	278	4219	2580	18886	21785	22503	4452	70206
Growth-based-visible categories	55	542	833	900	129	2459	1909	14846	17632	18994	3598	56979
Growth-based-invisible categories	80	465	483	583	149	1760	671	4040	4153	3509	854	13227
Livelihood-based categories	39	385	537	653	170	1784	390	3680	4315	8055	1732	18172
Total	174	1392	1853	2136	448	6003	2970	22566	26100	30558	6184	88378

The source of Table 1 is from this paper.

The statistics in Table 1 show that the number and investment amount of annual incremental PPP projects increased annually from 2014 to 2017. The reason for the sharp decrease in 2018 was the implementation of the PPP clear-out policy. There are significantly more growth-based PPP projects than livelihood-based PPP projects, and growth-based-visible PPP projects account for the majority of growth-based PPP projects. The intuitive analysis of the statistical results reveals that there is indeed a phenomenon of “emphasizing performance over livelihood” and “emphasizing aboveground over underground” in PPP investment.

#### 3.1.2 Local officials’ promotion indicators.

The paper involves data on the personal characteristics of local officials (municipal party secretaries and mayors) in 318 cities by searching local government websites. The data include tenure, gender, age, education and major. By matching the PPP project data (the year the PPP project entered the implementation phase) with the local official characteristic data (the year the local officials were in office), 920 growth-based PPP samples and 644 livelihood-based PPP samples were obtained. Referring to the literature [43,44], the tenure of officials is determined as follows: (1) If the official takes office before 30th June, the current year is considered tenure; otherwise, the following year is the first year of tenure. (2) If the official leaves office after 1st July, the current year is considered tenure; otherwise, the tenure ends in the previous year. (3) If more than one official takes office in the same year, the official who has the longest serving time in the current year is considered the local official of the current year. Each city matches with one mayor and one municipal party secretary, and the statistical information of the local officials’ tenure and age are shown in Table 2.

**Table 2:** Statistical information of local officials’ tenure and age.

Municipal party secretaries				Mayors			
Tenure (years)	Number of officials	Number percentage	Average age	Tenure (years)	Number of officials	Number percentage	Average age
1	376	33.97%	53.02	1	364	32.88%	51.67
2	273	24.66%	53.54	2	271	24.48%	51.85
3	273	24.66%	53.54	2	271	24.48%	51.85
4	137	12.38%	54.09	4	155	14.00%	51.98
5	75	6.78%	54.73	5	69	6.23%	53.03
6	31	2.80%	54.71	6	18	1.63%	53.44
7	8	0.72%	55.75	7	8	0.72%	53.13
8	7	0.63%	57.00	8	4	0.36%	54.75
9	4	0.36%	57.75	9	2	0.18%	53.50
				10	2	0.18%	54.50
				11	1	0.09%	56.00
Total	1107	100.00%	53.71	Total	1107	100.00%	51.92

The source of Table 2 is from this paper.

According to “the Interim Provisions on the Tenure of Office of Party and Government Leaders” and “the Decision on the Establishment of a Retirement System for Retired Cadres” issued by the General Office of the CPC Central Committee, local officials’ tenure is five years per term in China, and the average age of local officials remains 55. As local officials’ promotional speed is usually half a level at one time, local officials have to “run at a brisk pace” to reach their desired political target before retirement [45,46]. The statistics in Table 2 show that most local officials’ tenure is between one and four years, with only approximately 10% of local officials’ tenure being more than four years. The average age of mayors is approximately 51–52 years, whereas the average age of municipal party secretaries is approximately 53–54 years. Ji et al. [47] reported that local officials’ average tenure is 3–4 years, and the critical age is 54 or 55 years. Our statistical results are largely consistent with the findings of the literature.

Based on the statistical analysis in Table 2, it is predicted that local officials have fewer promotion incentives in the first two years of their tenure. However, starting in the third year, local officials in the second half of their tenure will have a stronger incentive to enhance their political performance that they tend to expand their growth-based PPP projects. The same situation occurs for local officials in the critical age range of 52–54.

#### 3.1.3 “Inverted U-shaped” curve.

**Fig 2:**
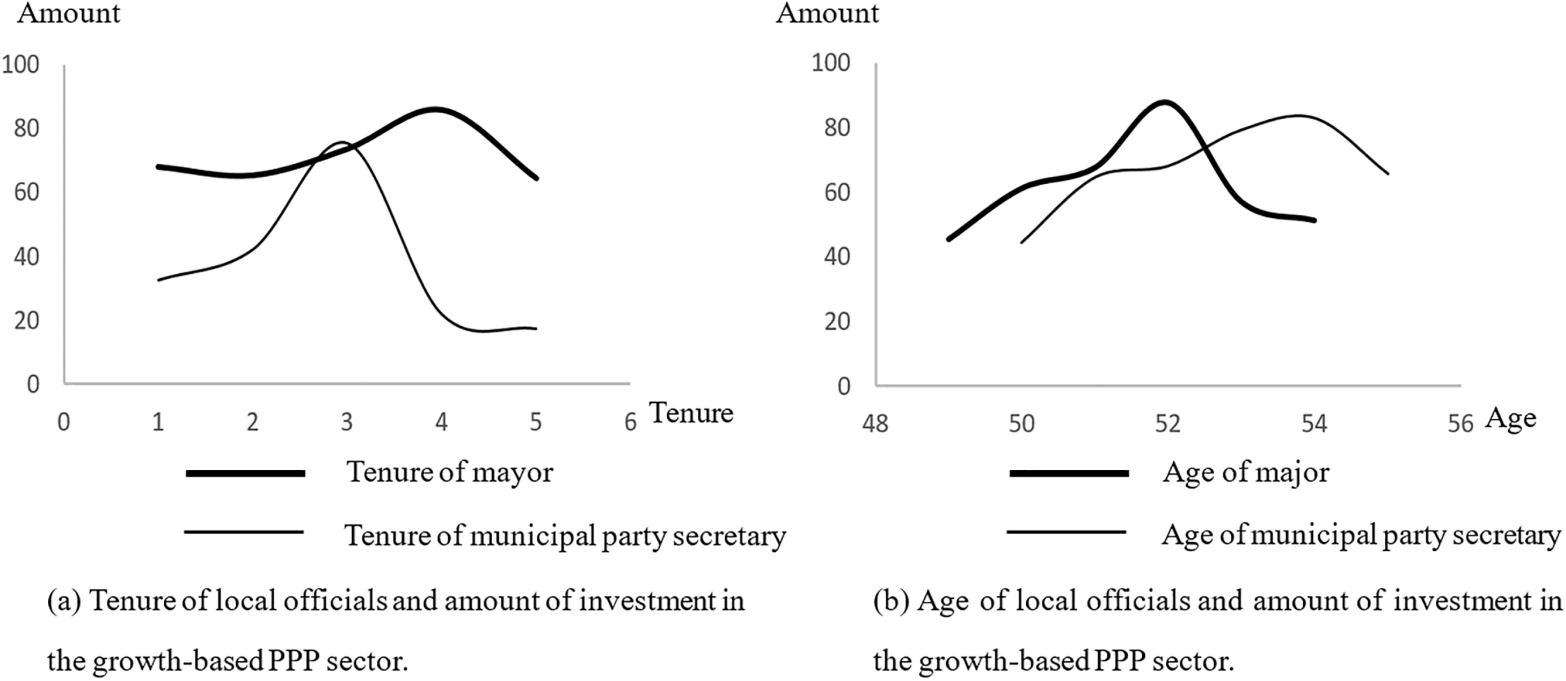
Local officials’ promotion incentives and investment amounts in growth-based PPP projects.

Fig 2 shows the relationship between local officials’ promotion incentives and the investment amount of growth-based PPP projects. This finding indicates that there is an inverted U-shaped curve between local officials’ promotion incentives and the investment amount of growth-based PPP projects. Fig 2(a) uses tenure as an indicator of promotion incentives and shows the change in the investment amount of growth-based PPP projects during local officials’ 5-year tenure. This finding indicates that local officials’ promotion incentives are relatively lower in the first 2 years of tenure, which is accompanied by a small amount of PPP investment. With respect to the critical point of promotion at 3–4 years of tenure, the PPP investment amount peaks and then begins to decrease. Fig 2(b) uses age as an indicator of promotion incentives and shows the change in the investment amount of growth-based PPP projects among municipal party secretaries aged 50–55 years and mayors aged 48–53 years. Because the mayors’ promotion threshold is 52 years old and the municipal party secretaries’ promotion threshold is 54 years old, the investment amount of growth-based PPP projects reaches a maximum at their threshold age.

#### 3.1.4 City characteristics.

We select per capita GDP, population, the proportion of secondary industry, fiscal revenue, the passenger volume of highways, the urban road area, the proportion of urban construction land, and the green coverage rate as indicators to describe city characteristics. The data are obtained from the Statistical Yearbooks of Chinese Cities. The missing data are collected manually from the Statistical Bulletin of National Economic and Social Development and government work reports released by each city’s government every year.

### 3.2 Variable selection

#### 3.2.1 Dependent variables.

The investment amount and number of annual incremental PPP projects are used as the dependent variables, all-samples are divided into growth-based categories and livelihood-based categories, and growth-based categories are divided into growth-based-visible categories and growth-based-invisible categories.

#### 3.2.2 Independent variables.

The tenure and age of local officials (mayors and municipal party secretaries) are used as explanatory variables. According to the statistical analysis, local officials’ tenure and age may have a threshold effect on PPP investment. In the heterogeneity analysis, we take 4 years as the mayor’s tenure threshold and 3 years as the municipal party secretary’s tenure threshold and take 52 years as the mayor’s age threshold and 54 years as the municipal party secretary’s age threshold to examine the heterogeneity of tenure and age.

#### 3.2.3 Controlled variables.

Considering other factors influencing PPP investment, we use PPP characteristics and city characteristics to control for PPP contract factors and macroeconomic environment factors and use local officials’ characteristics to control for the personal growth factors of mayors and municipal party secretaries.

**Fig 3:**
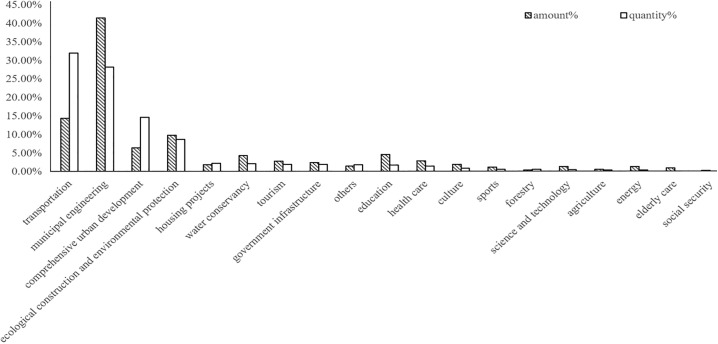
The percentage of each PPP category’s investment amount and quantity.

(1) PPP characteristics. As the allocation of control rights and the risk-sharing mechanism have important effects on PPP investment [48,49], we use the PPP return mechanism and cooperation period to control these two influencing factors. In general, by only entering the implementation phase, PPP projects can contribute to local officials’ performance. However, most PPP projects have a long preparation phase such that the year of initiation is not the same as the year of entry into the implementation phase. For example, one PPP project initiated in 2014 may have entered the implementation phase in 2015. If the local official who initiated the PPP project left office at the end of 2014, even if the local office has expended much effort on the PPP project, the PPP project helps little in his performance assessment and promotion. We set a dummy variable to control this situation: a value of 0 is assigned if the situation that the year of initiation is not the same as the year of entry into the implementation phase happened in any PPP project; otherwise, a value of 1 is assigned.
(2) Local officials’ characteristics. Three indicators, i.e., working experience in the central government department, gender, and education level, are used to control for local officials’ characteristics.
(3) City characteristics. As local financial capacity and local economic conditions influence PPP investment [50, 51], we set per capita GDP, population, the proportion of secondary industry, and fiscal revenue to control for city characteristics. As shown in Fig 3, among the PPP projects that have entered the implementation phase, four categories, namely, transportation, municipal engineering, comprehensive urban development, ecological construction and environmental protection, account for more than 10% of the PPP investment amount and quantity. A possible explanation for this phenomenon is that, influenced by the need for urban construction, cities have a greater demand for public transportation services, which leads to a large investment in these four PPP categories. We use the passenger volume of highways, the urban road area, the proportion of urban construction land, and the green coverage rate to control the impact of urban construction on PPP investment. All control variables use lagged one-period values.

Table 3 provides the definitions of all the variables.

**Table 3:** Definition description of all variables.

Variable type	Variable name	Variable symbols	Variable definitions
Dependent variables	PPP investment amount	*Capital*	Take the logarithm, amount of annual incremental PPP investment
	PPP investment quantity	*No*	Quantity of annual incremental PPP investment
Independent variables	Local officials’ tenure	*Tenure*	Tenure of mayor and municipal party secretary
	Local officials’ age	*Age*	Age of mayor and municipal party secretary
PPP characteristics	Difference in year	*Step*	If the situation that the year of initiation is not the same as the year of entry into the implementation phase happened in any PPP project in certain city, assigns values as 0. If the year of initiation is the same as the year of entry into the implementation phase in all PPP projects in certain city, assigns values as 1.
	Return mechanism	*Rs*	Dummy variables. If government payment occurs in the return mechanism of any PPP project in certain city, assigns values as 1. If there is no government payment in any PPP project, but user payment occurs in the return mechanism of any PPP project in certain city, assigns values as 2. If the return mechanism of all PPP projects is viability gap compensation, assigns values as 3.
	Cooperation period	*Duration*	Dummy variable. If there is any PPP project’s cooperation period over 30 (include 30) in certain city, assigns values as 3. If there is no PPP project’s cooperation period over 30, but there is any PPP project’s cooperation period between 20 and 30 in certain city, assigns values as 2. If the cooperation period of all PPP projects is less than 20, assigns values as 1.
local officials’ characteristics	Working experience	*Work*	If the local official have had working experience in the central government department, assigns values as 1, otherwise 0
	Gender	*Sexual*	Male is assigned value as 1 and female is assigned value as 0
	Education level	*Education*	Dummy variables. Below bachelor degree-0, bachelor degreen-1, master degree-2, doctor degree-3
City characteristics	Per capita GDP	*GDP*	take the logarithm, lagged one-period value
	Population	*Population*	Total population, take the logarithm, lagged one-period value
	Proportion of the secondary industry	*Industry*	Proportion of the secondary industry output value in total GDP, lagged one-period value
	Fiscal revenue	*Finance*	take the logarithm, lagged one-period value
	Passenger volume of highway	*HPT*	Number of passengers transported by highway, take the logarithm, lagged one-period value
	Urban road area	*Road*	Actual urban road area at the end of the current year, take the logarithm, lagged one-period value
	Proportion of urban construction land	*UC*	Proportion of urban construction land in total urban area, lagged one-period value
	Green coverage rate	*GCA*	Green coverage rate of built-up areas, lagged one-period value

The source of Table 3 is from this paper.

### 3.3 Model selection

We construct a two-way-fixed-effect panel model to test the impact of local officials’ promotion incentives on PPP investment. The regression model is shown in equations (1) and (2):


$${Capital}_{it}=\alpha +\beta_{\text{1}}{Tenure}_{it}+\beta_{\text{2}}{Tenure}_{it}^{\text{2}}+\beta_{\text{3}}{Age}_{it}+\beta_{\text{4}}{Age}_{it}^{\text{2}}+\gamma X_{it}+\lambda_{i}+\mu_{i}+\xi_{it}$$ (1)

$${No}_{it}=\alpha +\beta_{\text{1}}{Tenure}_{it}+\beta_{\text{2}}{Tenure}_{it}^{\text{2}}+\beta_{\text{3}}{Age}_{it}+\beta_{\text{4}}{Age}_{it}^{\text{2}}+\gamma X_{it}+\lambda_{i}+\mu_{i}+\xi_{it}$$ (2)

The $\;{Capital}_{it}$ and ${No}_{it}$ denote the amount and quantity of annual incremental PPP investment in the city *i* in the year *t*, respectively. PPP projects are divided into growth-based categories and livelihood-based categories, growth-based-visible ca*t*egories and growth-based-invisible categories. ${Tenure}_{it}$ and ${Age}_{it}$ denote the tenure and age of the mayor or municipal party secretary in the city *i* in the year *t*, respectively. ${Tenure}_{it}^{\text{2}}$ and ${Age}_{it}^{\text{2}}$ denote the square of tenure and age, respectively. $X_{it}$ represents a set of control variables, in which the variables of PPP characteristics and local officials’ characteristics take current da*t*a and the variables of city characteristics take lagged one-period data. $\lambda_{i}$ denotes individual fixed effects, $\mu_{i}$ denotes year-fixed effects, and $\xi_{it}$ denotes random error term.

## 4. Regression results analysis

### 4.1 Baseline regression analysis

Table 4 presents the baseline regression results of the impact of local officials’ promotion incentives on PPP investment. Table 4 (1) presents the regression results of the growth-based PPP categories. In Columns (1) and (2), the regression coefficients of tenure and age are both significant at the 1% level, indicating that the promotion incentives of the municipal party secretary have a significant positive effect on the growth-based PPP investment amount, thus verifying H1. In Columns (1) and (2), the regression coefficients of tenure squared and age squared are both significant at the 5% level, the joint regression coefficient between tenure and tenure squared is significant at the 5% level, and the joint regression coefficient between age and age squared is significant at the 10% level. This finding indicates that there is an inverted U-shaped curve relationship between the promotion incentives of the municipal party secretary and the growth-based PPP investment amount, thus verifying H2. In Columns (3) and (4), the regression coefficients of tenure and age are insignificant, indicating that the promotion incentives of the municipal party secretary have no significant positive effect on growth-based PPP investment quantity. In Columns (4) to (8), the promotion incentives of mayors do not have a significant effect on growth-based PPP investment. The regression results of Table 4 (2) show that neither the municipal party secretary’s promotion incentives nor the mayor’s promotion incentives have a significant effect on livelihood-based PPP investment, thus verifying H1a.

**Table 4:** Impact of local officials’ promotion incentives on PPP investment. (1). Growth-based PPP categories. (2). Livelihood-based PPP categories.

	Municipal party secretary				Mayor			
	Amount		Quantity		Amount		Quantity	
	(1)	(2)	(3)	(4)	(5)	(6)	(7)	(8)
${Tenure}_{it}$	0.0834*** (3.20)		0.1385 (0.94)		0.0582^*^ (1.82)		0.0956 (0.82)	
${Tenure}_{it}^{\text{2}}$	-0.0049^*^ (−1.97)		−0.0286 (−0.22)		−0.0138 (−1.33)		−0.0110 (−1.19)	
${Age}_{it}$		0.0389^**^ (2.38)		0.0739 (1.41)		0.0868 (0.37)		0.0057 (1.25)
${Age}_{it}^{\text{2}}$		-0.0095^**^ (−2.61)		−0.0103 (−1.38)		−0.0040^**^ (−2.43)		−0.0023 (−0.58)
*C*	3.5783^**^ (2.48)	1.4874^*^ (1.97)	−2.3844 (−1.46)	−1.5763^*^ (−1.81)	1.9965^**^ (2.36)	1.5786^**^ (2.54)	−6.2378 (−0.93)	−5.2387 (−1.44)
*Controls*	Yes	Yes	Yes	Yes	Yes	Yes	Yes	Yes
P-value	0.1745	0.2850	0.0734	0.1955	0.3859	0.1898	0.0374	0.2895
Sample size	920	920	920	920	920	920	920	920
Adjustment of *R*^*2*^	0.2358	0.2177	0.1680	0.1694	0.2399	0. 2527	0.1539	0.1542
${Tenure}_{it}$	0.1849 (1.52)		0.0682 (0.39)		0.0673 (0.48)		0.0629 (0.67)	
${Tenure}_{it}^{\text{2}}$	-0.024 (−1.69)		−0.0083 (−1.52)		−0.0284 (−0.79)		−0.0073 (−0.30)	
${Age}_{it}$		0.0021 (0.18)		0.0044 (0.27)		0.0012 (1.02)		0.0304 (0.03)
${Age}_{it}^{\text{2}}$		-0.0010 (−0.76)		−0.0018 (−1.26)		−0.0006 (−1.13)		−0.0087 (−0.37)
*C*	7.2384 (1.07)	6.1329^**^ (2.56)	−12.5683 (−0.39)	−14.2389^*^ (−1.88)	6.2984 (0.32)	8.9423^*^ (2.01)	−14.5986 (−0.95)	−14.6895 (−1.37)
*Controls*	Yes	Yes	Yes	Yes	Yes	Yes	Yes	Yes
P-value	0.6382	0.5032	0.6735	0.5933	0.4923	0.3758	0.3874	0.2873
Sample size	644	644	644	644	644	644	644	644
Adjustment of *R*^*2*^	0.1845	0.0593	0.1957	0.1567	0.0839	0.0936	0.2584	0.2267

Notes: P-values indicate the P-value of the joint significance test of promotion incentive (tenure or age) and promotion incentive squared. ***, ** and * indicate the significance at the 1%, 5% and 10% levels, respectively.

The source of Table 4 is from this paper.

The regression results indicate that when promotion is motivated by the tenure and age of the municipal party secretary, the PPP investment has the characteristic of “emphasizing performance over livelihood”. Compared with growth-based PPP investment, local officials’ promotion incentives have less of an impact on livelihood-based PPP investment. Compared with investment quantity, the municipal party secretary has a stronger incentive to increase the growth-based PPP investment amount. When the tenure and age of the municipal party secretary reach a certain threshold, the annual incremental growth-based PPP investment amount reaches a maximum, and the mayor’s promotion incentives have no significant effect on PPP investment. This suggests that, driven by promotion incentives, the municipal party secretary tends to increase the growth-based PPP investment amount [10], whereas the mayor does not give attention to PPP investment.

### 4.2 Endogenous tests

#### 4.2.1 Endogenous reverse causality.

The decision of whether to set up PPP projects is made by the local government but not the central government, and PPP investment is not a direct indicator of the central government’s ability to assess local government performance [3]. Therefore, there is a minor possibility that PPP investment has a direct and significant effect on the promotion of local officials and that reverse causality relatively does not exist.

#### 4.2.2 Endogenous missing variables.

We note that several endogenous missing variables influence PPP investment in this paper.

Local officials with a specific major or education background are more likely to focus on PPP categories related to their major. For example, local officials who major in science and technology are more likely to take office in cities with low levels of science and technology [4]. They give more attention to the city’s science and technology industry, and they are more willing to increase PPP investment in this category. To solve the endogenous problems caused by local offices’ education background, we add a dummy variable named “*consistence*” as the control variable. If a local officials’ major is consistent with the PPP category, the dummy variable is equal to 1; otherwise, it is 0.

In addition, the development of a specific industry in a city influences PPP investment. Given the need to close the gap in development shortcomings, cities with a lower level of science and technology are more likely to attract PPP investment in the category of science and technology [4]. We add two control variables named “*gap 1*” and “*gap2*”. The data of “*gap 1*” are the secondary industry output values reflecting the development of the industry that corresponds to the city’s attraction to the PPP growth-based categories. The data of “*gap 2*” are the third industry output value reflecting the development of the service industry, which corresponds to the city’s attraction to the PPP livelihood-based categories.

The regression results are shown in Table 5. According to the regression results, the conclusions of the endogenous test remain consistent with the baseline conclusions.

**Table 5:** The Endogenous tests. (1). Growth-based PPP categories. (2). Livelihood-based PPP categories.

	Municipal party secretary				Mayor			
	Amount		Quantity		Amount		Quantity	
	(1)	(2)	(3)	(4)	(5)	(6)	(7)	(8)
${Tenure}_{it}$	0.0785*** (3.64)		0.1064 (1.47)		0.03910 (1.04)		0.0940 (1.48)	
${Tenure}_{it}^{\text{2}}$	-0.0032** (−2.25)		−0.0193 (−0.48)		−0.0027^*^ (−1.83)		−0.0104 (−1.46)	
${Age}_{it}$		0.0419*** (3.07)		0.0659^*^ (1.76)		0.0222 (1.02)		0.0158 (0.39)
${Age}_{it}^{\text{2}}$		-0.0028** (−2.14)		−0.0050 (−1.57)		−0.0058 (−1.15)		−0.0009 (−0.78)
C	1.5693** (2.12)	2.4134^***^ (3.19)	−6.4035 (−0.29)	−5.1904^**^ (−2.24)	0.6367 (0.05)	3.0570 (0.23)	−8.4285 (−0.38)	−7.1185 (−0.32)
Controls	Yes	Yes	Yes	Yes	Yes	Yes	Yes	Yes
P-value	0.0382	0.0501	0.1842	0.1139	0.2805	0.3923	0.3188	0.2246
Sample size	920	920	920	920	920	920	920	920
Adjustment of R2	0.1700	0.1726	0.5944	0.5917	0.1743	0.1776	0.6004	0.5987
${Tenure}_{it}$	0.0368 (0.70)		0.0450 (0.56)		0.04561 (1.04)		0.0799 (1.22)	
${Tenure}_{it}^{\text{2}}$	-0.0045 (−1.06)		−0.0033 (−1.29)		−0.0058 (−0.91)		−0.0068 (−0.21)	
${Age}_{it}$		0.0004 (0.02)		0.01253 (0.36)		0.0020 (0.07)		0.0409 (0.81
${Age}_{it}^{\text{2}}$		-0.0001 (−0.45)		−0.0006 (−0.89)		−0.0005 (−1.12)		−0.0022 (−1.42)
*C*	15.9540 (0.93)	15.9968 (0.95)	−37.8561^*^ (−1.71)	−37.9707^*^ (−1.70)	11.7346 (0.69)	12.2809 (0.69)	−37.8143^*^ (−1.81)	−35.2240 (−1.64)
*Controls*	Yes	Yes	Yes	Yes	Yes	Yes	Yes	Yes
P-value	0.5839	0.4932	0.6185	0.4403	0.7782	0.5311	0.2146	0.3471
Sample size	644	644	644	644	644	644	644	644
Adjustment of *R*^*2*^	0.0490	0.0407	0.3236	0.3290	0.1143	0.1111	0.3343	0.3395

Notes: ***, ** and * indicate the significance at the 1%, 5% and 10% levels, respectively.

The source of Table 5 is from this paper.

### 4.3 Discussion on growth-based PPP sub-categories

We divide growth-based PPP categories into the growth-based-visible categories and the growth-based-invisible categories to test the impact of municipal party secretary’s promotion incentives on the investment amount of the two sub-categories. According to the regression results in Table 6, the impact of municipal party secretary’s promotion incentives on the growth-based-visible PPP investment amount is highly significant. Although the impact of municipal party secretary’s promotion incentives on the growth-based-invisible PPP investment amount also shows an “inverted U-shaped” curve, the significance level and the regression coefficients decrease. The regression results indicate that, driven by promotion incentives, compared with growth-based-invisible PPP categories, municipal party secretary pays more attention to the growth-based-visible PPP categories, and the PPP investment shows the characteristic of “emphasizing aboveground over underground”, thus verifying H1b.

**Table 6:** Impact of municipal party secretary’s promotion incentives on the growth-based PPP sub-categories’ investment amount.

	Growth-based-visible categories		Growth-based-invisible categories	
	(1)	(2)	(3)	(4)
${Tenure}_{it}$	0.0924*** (2.91)		0.0456 (1.12)	
${Tenure}_{it}^{\text{2}}$	-0.0087** (−2.32)		−0.0023^**^ (−2.37)	
${Age}_{it}$		0.1219** (2.22)		0.0312^**^ (2.22)
${Age}_{it}^{\text{2}}$		-0.0123*** (−3.17)		−0.0055^**^ (−2.23)
*C*	3.3086^***^ (3.44)	2.9190^***^ (3.62)	8.8785^**^ (2.27)	4.7609^**^ (2.07)
*Controls*	Yes	Yes	Yes	Yes
P-value	0.0145	0.0389	0.0344	0.0872
Sample size	747	747	776	776
Adjustment of *R*^*2*^	0.1729	0.1776	0.1657	0.1031

Notes: ***, ** and * indicate the significance at the 1%, 5% and 10% levels, respectively.

The source of Table 6 is from this paper.

### 4.4 The mayors’ age threshold

The regression analysis above reveals that the impact of a mayor’s promotion incentives on the amount of PPP investment is not significant. However, the regression coefficient of the mayor’s tenure squared on the growth-based PPP investment amount is significant at the 10% level. The possible reason for this result is that, according to the curve in Fig 1, considering that 52 is the mayor’s age threshold for promotion [45–47], compared with mayors under 52 years of age and mayors over 52 years of age, the impacts of their tenure incentives on the amount of PPP investment differ [41,42]. A mayor who is over the age threshold faces a situation in which the likelihood of promotion is significantly lower, which causes a weakened promotion incentive. To examine the heterogeneity of mayor age, we distinguish mayors into young mayors and senior mayors and define young mayors as mayors under 52 years of age and senior mayors as mayors over 52 years of age. We match the PPP samples to young mayors and senior mayors and perform regressions on these two types of samples.

Table 7 reports the regression results of the impact of a mayor’s tenure incentive with an age threshold on the PPP investment amount. For the sample of young mayors, the regression coefficients between tenure and investment amount in the growth-based PPP categories and the growth-based-visible PPP categories are both significant at the 10% level. As the joint regression coefficients between tenure and tenure squared are significant, an inverted U-shaped relationship exists. The regression coefficients for the livelihood-based PPP categories and the growth-based-invisible PPP categories are insignificant. For the sample of senior mayors, all regression coefficients are insignificant. The regression results indicate that young mayors and senior mayors maintain different levels of promotion incentives. As young mayors have more opportunities to obtain promotions, they are willing to improve their political performance and increase their investment in PPP projects.

**Table 7:** Impact of mayor’s tenure incentive with age threshold on the PPP investment amount.

PPP categories	Young mayors				Senior mayors			
	Growth-based	Livelihood-based	Growth-based-visible	Growth-based-invisible	Growth-based	Livelihood-based	Growth-based-visible	Growth-based-invisible
${Tenure}_{it}$	0.0162* (1.81)	0.0196 (0.82)	0.1054^*^ (1.75)	0.1303 (0.39)	−0.0819 (−1.38)	−0.0165 (−0.51)	0.0231 (1.25)	−0.0697 (−1.34)
${Tenure}_{it}^{\text{2}}$	-0.0024* (−1.79)	−0.0045 (−1.08)	−0.0374^**^ (−2.37)	−0.0642 (−1.07)	0.0044 (1.12)	0.0024 (0.44)	−0.0028 (−0.36)	0.0012 (0.66)
*C*	9.1960^**^ (2.19)	16.0569 (0.60)	2.1114^*^ (1.76)	5.0183 (1.51)	−6.5767 (−1.05)	−0.9196 (−1.03)	1.5893^*^ (1.83)	10.9211 (1.49)
*Controls*	Yes	Yes	Yes	Yes	Yes	Yes	Yes	Yes
P-value	0.0749	0.1095	0.0956	0.1968	0.2927	0.6138	0.5807	0.2070
Sample size	482	317	438	194	245	328	221	180
Adjustment of *R*^*2*^	0.1985	0.1528	0.1382	0.1227	0.1896	0.1985	0.1572	0.1663

Notes: ***, ** and * indicate the significance at the 1%, 5% and 10% levels, respectively.

The source of Table 7 is from this paper.

### 4.5 Robustness test

In addition to age and tenure, the local economic and social situation are also taken into account when local officials consider promotion. Referring to the literature [3], we construct a promotion incentive indicator as *Eco-Social* containing the GDP growth rate, public finance surplus, unemployment rate, age, and tenure for a robustness test.

Taking the GDP growth rate as an example, the calculation is as follows:


$$P_{n}=\frac{\tilde{{GDP}_{n}}}{\sum_{\text{1}}^{N}\tilde{{GDP}_{n}}}$$ (3)

$$\begin{array}{c} {\mathrm{\backslash itDelta}GDP}_{M}=\sum_{\text{1}}^{N}P_{n}\times {\mathrm{\backslash itDelta}GDP}_{n} \end{array}$$ (4)

$${Eco-Social}_{GDP}=\left\{ \begin{array}{c} @l\text{1},\;\;\;if\;{\mathrm{\backslash itDelta}GDP}_{n}<{\mathrm{\backslash itDelta}GDP}_{M}\\ \text{0},\;\;\;if\;{\mathrm{\backslash itDelta}GDP}_{n}>{\mathrm{\backslash itDelta}GDP}_{M} \end{array}\right.\;$$ (5)

Province *M* contains a total of *N* cities, *N* = 1, 2,..., *n*. $P_{n}$ is the calculated weight. $\tilde{{GDP}_{n}}$ is the GDP per capita of city *n*. ${\mathrm{\backslash itDelta}GDP}_{n}$ is the GDP growth rate of city *n*. ${\mathrm{\backslash itDelta}GDP}_{M}$ is the calculated GDP growth rate of province *M*. When the local GDP growth rate of a city is less than the GDP growth rate of the province to which the city belongs, the indicator *Eco-Social*_*GDP*_ of the GDP growth rate is assigned a value of 1. Otherwise, *Eco-Social*_*GDP*_ is assigned a value of 0. This calculation uses the same method to assign the value of the indicator *Eco-Social*_*pfs*_ of the public finance surplus. For the unemployment rate, when the local unemployment rate of a city is less than the unemployment rate of the province to which the city belongs, the indicator *Eco-Social*_*ur*_ of the unemployment rate is assigned a value of 0. Otherwise, the indicator *Eco-Social*_*ur*_ is assigned a value of 1.

According to the statistical analysis above, both local officials’ tenure and age have an “inverted U-shaped” curve relationship with the growth-based PPP investment amount. When the municipal party secretary reaches the age of 54 and the 3^rd^ year of their term, the growth-based PPP investment amount peaks. Therefore, when the municipal party secretary’s age is 53 or 54 years, the indicator *Eco-Social*_*age*_ of age is assigned a value of 1. Otherwise, the indicator *Eco-Social*_*age*_ is assigned a value of 0. When the municipal party secretary’s tenure is in the 2^nd^ or 3^rd^ year, the indicator *Eco-Social*_*tenure*_ of tenure is assigned a value of 1. Otherwise, the indicator *Eco-Social*_*tenure*_ is assigned a value of 0.

Summing the values of the five indicators, *Eco-Social*_*GDP*_, *Eco-Social*_*pfs*_, *Eco-Social*_*ur*_, *Eco-Social*_*age*_ and *Eco-Social*_*tenure*_, the municipal party secretary’s comprehensive promotion incentive indicator *Eco-Social* is obtained, with a value ranging from 0 to 5. The higher the total value is, the greater the level of the municipal party secretary’s promotion incentive. We replace the original promotion incentive indicators—age and tenure—with the comprehensive promotion incentive indicator *Eco-Social* and perform a regression on the municipal party secretary’s new promotion incentive and the PPP investment amount.

The regression results are shown in Table 8. There is a significant positive correlation between the municipal party secretary’s new promotion incentive and the amount of PPP investment. The promotion incentive positively and significantly influences the investment amount of growth-based PPP categories, and growth-based-visible PPP categories indicate that the higher the level of promotion incentive is, the more willing the municipal party secretary is to increase the investment amount of growth-based PPP categories and growth-based-visible PPP categories. These results verify our above viewpoint that, driven by the promotion incentive, the distribution of PPP investment has the characteristics of “emphasizing performance over livelihood” and “emphasizing aboveground over underground”.

**Table 8:** Robustness test for the impact of municipal party secretary’s comprehensive promotion incentive on PPP investment.

	PPP categories		Growth-based PPP categories	
	Growth-based	Livelihood-based	Growth-based-visible	Growth-based-invisible
*Eco-Social*	0.0753^**^ (2.28)	0.0608 (1.25)	0.0969^***^ (3.26)	0.0108 (0.75)
*C*	4.3078^**^ (2.12)	9.3786 (1.43)	3.5877^***^ (3.95)	0.4894^*^ (1.81)
*Controls*	Yes	Yes	Yes	Yes
Sample size	920	644	747	776
Adjustment of *R*^*2*^	0.1702	0.0835	0.1752	0.2385

Notes: ***, ** and * indicate the significance at the 1%, 5% and 10% levels, respectively.

The source of Table 8 is from this paper.

According to the analysis of the regression results, the conclusions of the robustness test remain consistent with the baseline conclusions.

Since 2018, the supervision of PPP projects has become stricter, and there is less chance for local officials to aggressively expand their PPP investment. As the robustness test selects PPP samples from 2014 to 2018, it is unable to test the influence of local officials’ promotion incentives on PPP investment after the implementation of strict supervision. We now expand the sample period to 2023 to test the robustness of the influence.

The regression results are shown in Table 9. There is a significant positive correlation between the municipal party secretary’s promotion incentive and growth-based PPP investment. Compared with the baseline regression results, although the coefficient values decrease, the significance of the values basically remains the same. According to the regression results, the conclusions of the robustness test remain consistent with the baseline conclusions.

**Table 9:** Robustness test of growth-based PPP categories from 2014 to 2023.

	Municipal party secretary				Mayor			
	Amount		Quantity		Amount		Quantity	
	(1)	(2)	(3)	(4)	(5)	(6)	(7)	(8)
${Tenure}_{it}$	0.0542*** (3.85)		0.0894 (1.56)		0.0183 (0.68)		0.0284 (1.23)	
${Tenure}_{it}^{\text{2}}$	-0.0058** (−2.31)		−0.0126 (−1.17)		−0.0025 (−1.61)		−0.0167^*^ (−1.78)	
${Age}_{it}$		0.0239^**^ (2.26)		0.0279^*^ (1.82)		0.0319 (0.76)		0.0106 (1.44)
${Age}_{it}^{\text{2}}$		-0.0072** (−2.09)		−0.0031^**^ (−2.14)		−0.0072 (−0.98)		−0.0072 (−1.27)
*C*	2.8546^***^ (3.93)	4.4962^*^ (1.90)	−0.9561^**^ (−2.34)	−0.3524^*^ (−1.88)	6.4652 (0.56)	1.4656 (1.26)	−2.5652 (−0.84)	−0.4865 (−0.98)
*Controls*	Yes	Yes	Yes	Yes	Yes	Yes	Yes	Yes
P-value	0.1254	0.1109	0.2538	0.2466	0.2671	0.2895	0.2385	0.2745
Sample size	1813	1813	1813	1813	1813	1813	1813	1813
Adjustment of *R*^*2*^	0.2013	0.2118	0.5723	0.5810	0.2076	0.2142	0.5764	0.5883

Notes: ***, ** and * indicate the significance at the 1%, 5% and 10% levels, respectively.

The source of Table 9 is from this paper.

## 5. Conclusions

The PPP model is widely applied in many industries, such as transportation, municipal engineering, health care, ecology and environmental protection, to relieve local government financial pressure and improve the efficiency of public service supply. However, the distributions of PPP quantity and investment amount are uneven in different industries in China. We take political economic theory as the theoretical foundation, conduct studies on the political reasons for PPP project investment mismatch from a politically performance-driven perspective, and determine the negative effect of local officials’ promotion incentives. The main conclusions are as follows:

First, a municipal party secretary’s promotion incentives have a significantly positive effect on growth-based PPP categories and growth-based-visible PPP categories. Driven by the municipal party secretary’s promotion incentive of tenure and age, there is a phenomenon of “emphasizing performance over livelihood” and “emphasizing aboveground over underground” in PPP investment.

Second, there is an “inverted U-shaped curve” relationship between the municipal party secretary’s promotion incentives and the growth-based PPP investment amount, as well as the growth-based-visible PPP investment amount. At the beginning of the tenure of the municipal party secretary, the amounts of the two types of PPP investment increase. It peaks at the 3^rd^ year of tenure and then decreases. The same changing trend of the PPP investment amount exists in the municipal party secretary’s age incentive, and the age at which the PPP investment amount reaches the peak is 54.

Third, the impact of mayors’ age on PPP investment is heterogeneous: 52 is the mayors’ age threshold for promotion. Young mayors who are under 52 years old have a stronger tenure incentive to increase growth-based PPP investment amounts and growth-based-visible PPP investment amounts, leading to uneven PPP investment. A senior mayor who is over 52 years old has a decreased tenure incentive, as their promotion opportunity decreases. The senior mayor’s tenure incentive has an insignificant effect on PPP investment.

The findings give empirical evidences to support the viewpoint that there is connection between PPP investment and local officials [52]. Different from the previous research that focuses more on local government [23–25,53–54] and PPP contract [15–21,55–56], this paper strongly point out that local officials’ performance incentive significantly affect PPP investment and analyzes the reasons for the uneven investment in PPP projects. Though Wang and Yu [57] reached the same conclusion as our findings, the official indicators in their research were from the data of mayor only, which means it ignored the influences of the party secretary. Our paper takes the mayor and party secretary both into consideration that makes up the missing.

## 6. Recommendations

Local officials’ promotion incentives are an important factor of uneven PPP investment that distorts the mismatch of public resources. In the robustness test, we find that after expanding the sample period to 2023, the influence of local officials’ promotion incentives on growth-based PPP investment decreases, which provides some insight into solving the problem of distortion. First, the central government could standardize the PPP contract to perfect the PPP access mechanism. The PPP contract could strictly stipulate the contract subjects, financing models, capital composition and payment mechanisms to avoid the risk of disrupting the competitive market. Second, the central government could make a “positive list and negative list” to build a PPP withdrawal mechanism. The PPP projects in the positive list are the excellent examples for other projects to learn from, while the PPP projects in the negative list are with some problems. Based on the severity of the problems, it could classify the PPP projects in the negative list into five grades, and ask for rectification within a specific time limit. Otherwise, the projects would be kicked out from PPP projects. Third, the central government could optimize the appraisal mechanism for local officials and design multiple appraisal indicators to induce good promotion competition. Those appraisal indicators focus on the long-term political performance and people’s livelihood that have little connection with local economy. As external powers, the public, society and third-party organizations could be induced into the appraisal mechanism. Their comments on local government take a significant role in the local officials’ promotion procedure.

There are also several limitations in this study. First, we divide PPP projects into growth-based categories and livelihood-based categories on the basis of the level 1 classification catalog of the projects. The regression results would be more credible if we have divided PPP projects based on their level 2 or 3 classification catalog. Second, we do additional research on growth-based PPPs but omit a discussion of livelihood-based PPPs. Third, we set some control variables to control for the extra influence of the central policies. This paper would become more substantial if we have conducted more in-depth research on those policies.

Based on the findings and limitations, we can implement future research from several aspects. First, focusing on the sub-catalogue of the PPP project provides a more detailed explanation of the influence of local officials’ promotion incentives on PPP investment. Second, the PPP strict supervision policies of 2018 provide a quasi-natural experiment to test the influence of supervision on local officials’ promotion incentives. As the central government pays more attention to the people’s livelihood issues in recent years, the future research could focus on the sub-catalogue of the PPP project on education, culture, elderly care, environmental protection, health care. Using the PPP regulatory policy which implemented in 2018 as quasi-natural experimental scene, it could take DID model as potential methodology to test the influence of local officials’ promotion incentives on the investment of livelihood-based PPP categories. The comparison between the influence of local officials’ promotion incentives on PPP investment before the implementation of supervision policies and the influence after the implementation of supervision policies provides evidence of an empirical test of the effect of strict supervision. Third, future studies could focus on optimizing the appraisal mechanism, which is beneficial for decreasing the negative influence of local officials’ promotion incentives on PPP investment. In recent years, Chinese central government has taken several steps to optimize the appraisal mechanism. It would be a good chance to test the validity of the optimized appraisal mechanism. DID model can be used to test the validity of the optimized appraisal mechanism, and the mediation effect model can be used to dig out the direct effect and indirect effect to local officials’ promotion incentives on PPP investment.
